# *Notes* *from the Field:* Long COVID and Significant Long COVID–Associated Activity Limitation Among Adults, by Jurisdiction — United States, 2023

**DOI:** 10.15585/mmwr.mm7350a2

**Published:** 2024-12-19

**Authors:** Nicole D. Ford, Abraham Agedew, Alexandra F. Dalton, Caroline Pratt, Cria O. Gregory, Sharon Saydah

**Affiliations:** ^1^Coronavirus and Other Respiratory Viruses Division, National Center for Immunization and Respiratory Diseases, CDC; ^2^General Dynamics Information Technology, Falls Church, Virginia.

**Summary**

**What is already known about this topic?**

Long COVID might limit a person’s ability to carry out day-to-day activities.

**What is added by this report?**

In 2023, 6.4% (95% CI = 6.3–6.6%) of the noninstitutionalized U.S. adults nationwide were experiencing Long COVID when surveyed. Among adults with current Long COVID, age- and sex-standardized prevalences of reporting significant Long COVID–associated activity limitation ranged from 12.8% in the District of Columbia to 29.4% in Puerto Rico. Among the seven jurisdictions with current Long COVID prevalence of ≥8.0%, Idaho, Puerto Rico, and West Virginia were also in the highest prevalence quintile for significant Long COVID–associated activity limitation.

**What are the implications for public health practice?**

These findings support the ongoing importance of tools to reduce risk for Long COVID, including vaccination. Jurisdiction-specific estimates might guide policy, planning, or programming to support U.S. adults reporting significant Long COVID–associated activity limitation.

## Introduction

Long COVID is a chronic condition comprising a wide range of symptoms and conditions lasting ≥3 months after SARS-CoV-2 infection (*1*). Frequently reported symptoms include fatigue that interferes with daily life, difficulty thinking or concentrating, cough, and heart palpitations.* One in four U.S. adults with Long COVID report significant activity limitations (*2*). Few representative subnational estimates are available to support jurisdiction-specific policy, planning, or programming for Long COVID.

## Investigation and Outcomes

CDC analyzed data from the 2023 Behavioral Risk Factor Surveillance System (BRFSS), a large, population-based cross-sectional survey of noninstitutionalized U.S. adults aged ≥18 years (*3*). BRFSS samples participants using random-digit–dialing of mobile and landline telephones.^†^ Self-reported age, sex, previous COVID-19 illness, current Long COVID, and significant activity limitation due to Long COVID were ascertained via telephone interview. Current Long COVID was defined as self-report of any symptoms lasting ≥3 months at the time of interview that were not present before having COVID-19. Significant activity limitation due to Long COVID was defined as the presence of symptoms reducing ability to carry out day-to-day activities “a lot” compared with such ability during the time before having COVID-19. CDC estimated weighted age- and sex-standardized prevalences with 95% CIs of current Long COVID among all adults and significant activity limitation due to Long COVID among adults experiencing Long COVID nationwide, in 48 states,^§^ the District of Columbia (DC), Guam, Puerto Rico, and the U.S. Virgin Islands. CDC standardized all estimates to the 2020 U.S. Census Bureau population of civilian, noninstitutionalized adults using sex-specific weights by age group for persons aged 18–44, 45–64, and ≥65 years. Analyses were conducted using SAS-callable SUDAAN (version 9.4; RTI International) to account for complex survey design. Prevalence estimates were divided into quintiles to create prevalence maps. This activity was reviewed by CDC, deemed not research, and was conducted consistent with applicable federal law and CDC policy.^¶^

## Preliminary Conclusions and Actions

During 2023, 6.4% (95% CI = 6.3%–6.6%) of noninstitutionalized U.S. adults nationwide were experiencing Long COVID when surveyed. The weighted age- and sex-standardized prevalence of current Long COVID ranged from 2.9% (95% CI = 1.7%–5.1%) in the U.S. Virgin Islands to 9.7% (95% CI = 8.7%–10.9%) in West Virginia (Supplementary Table, https://stacks.cdc.gov/view/cdc/174567). Among adults with current Long COVID, 19.8% (95% CI = 18.9%–20.8%) reported significant activity limitations due to their symptoms. The weighted age- and sex-standardized prevalence of significant activity limitation because of Long COVID among U.S. jurisdictions ranged from 12.8% (95% CI = 7.8%–20.3%) in DC to 29.4% (95% CI = 23.6%–36.0%) in Puerto Rico (Figure). Among the seven jurisdictions with current Long COVID prevalence of ≥8.0%, Idaho, Puerto Rico, and West Virginia were also in the highest prevalence quintile for significant Long COVID–associated activity limitation.

**FIGURE:**
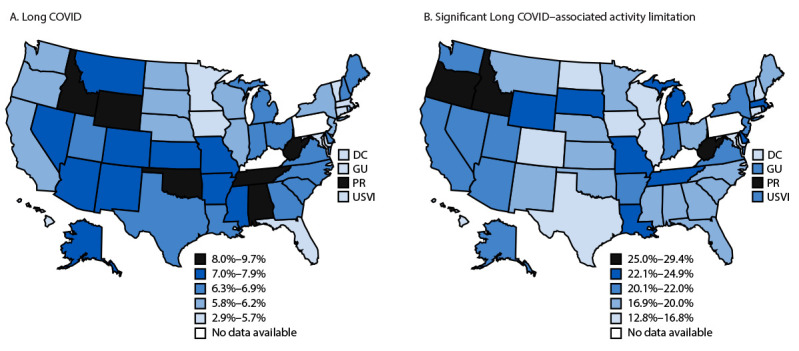
Prevalences of current Long COVID (A) and significant Long COVID–associated activity limitation (B) among adults, by jurisdiction — Behavioral Risk Factor Surveillance System, United States, 2023*^,†^ **Abbreviations:** DC = District of Columbia; GU = Guam; PR = Puerto Rico; USVI = U.S. Virgin Islands. * Prevalence estimates are weighted to account for complex sampling and standardized to the U.S. civilian, noninstitutionalized population based on 2020 U.S. Census Bureau population. Sex-specific weights by age group were applied for ages 18–44, 45–64, and ≥65 years. ^†^ Denominators for prevalence estimates of current Long COVID are the total adult population. Denominators for prevalence estimates for significant activity limitation are the number of adults with current Long COVID.

These findings support the ongoing importance of tools to reduce the risk for Long COVID, including vaccination. Adults with Long COVID, particularly those with significant Long COVID–associated activity limitation, might require additional supports to aid recovery, such as health care resources and workplace accommodations (*4*,*5*). These estimates might help guide jurisdiction-specific policy, planning, or programming to support U.S. adults reporting Long COVID–associated limitations.

## Footnotes

* https://www.cdc.gov/covid/long-term-effects/long-covid-signs-symptoms.html^†^ In 2023, BRFSS surveyed 433,323 adults. The median survey response rate for all participating jurisdictions in 2023 was 44.7% and ranged from 21.7% to 63.1%. https://www.cdc.gov/brfss/annual_data/2023/pdf/2023-DQR-508.pdf

§ In 2023, Kentucky and Pennsylvania were unable to collect sufficient data to meet the minimum requirements to be included in the BRFSS public data set.

¶ 45 C.F.R. part 46.102(l)(2), 21 C.F.R. part 56; 42 U.S.C. Sect. 241(d); 5 U.S.C. Sect. 552a; 44 U.S.C. Sect. 3501 et seq.
